# Characterization of Novel Transcripts in Pseudorabies Virus

**DOI:** 10.3390/v7052727

**Published:** 2015-05-22

**Authors:** Dóra Tombácz, Zsolt Csabai, Péter Oláh, Zoltán Havelda, Donald Sharon, Michael Snyder, Zsolt Boldogkői

**Affiliations:** 1 Department of Medical Biology, Faculty of Medicine, University of Szeged, Somogyi B. u. 4., Szeged H-6720, Hungary; E-Mails: tombacz.dora@med.u-szeged.hu (D.T.); csabai.zsolt@med.u-szeged.hu (Z.C.); olah.peter@med.u-szeged.hu (P.O.); 2 Agricultural Biotechnology Center, Institute for Plant Biotechnology, Plant Developmental Biology Group, Szent-Györgyi A. u. 4, Gödöllő H-2100, Hungary; E-Mail: havelda@abc.hu; 3 Department of Genetics, School of Medicine, Stanford University, 300 Pasteur Dr., Stanford, CA 94305-5120, USA; E-Mails: dsharon@stanford.edu (D.S.); mpsnyder@stanford.edu (M.S.)

**Keywords:** non-coding RNA, pseudorabies virus, herpesvirus, RNA sequencing, DNA replication

## Abstract

In this study we identified two 3′-coterminal RNA molecules in the pseudorabies virus. The highly abundant short transcript (CTO-S) proved to be encoded between the *ul21* and *ul22* genes in close vicinity of the replication origin (OriL) of the virus. The less abundant long RNA molecule (CTO-L) is a transcriptional readthrough product of the *ul21* gene and overlaps OriL. These polyadenylated RNAs were characterized by ascertaining their nucleotide sequences with the Illumina HiScanSQ and Pacific Biosciences Real-Time (PacBio RSII) sequencing platforms and by analyzing their transcription kinetics through use of multi-time-point Real-Time RT-PCR and the PacBio RSII system. It emerged that transcription of the CTOs is fully dependent on the viral transactivator protein IE180 and CTO-S is not a microRNA precursor. We propose an interaction between the transcription and replication machineries at this genomic location, which might play an important role in the regulation of DNA synthesis.

## 1. Introduction

Pseudorabies virus (PRV), an alphaherpesvirus related to the human pathogens herpes simplex virus (HSV) and varicella-zoster virus, infects a wide range of mammalian species, including experimental rodents and pigs, the reservoir of the virus. PRV is commonly used in investigations of the molecular pathogenesis of herpesviruses [1,2], for the mapping of neural circuits [3,4,5] and for the delivery of genetically encoded fluorescence activity markers to the central nervous system [6] and cardiomyocytes [7]. During the past few years, a large variety of non-coding RNAs (ncRNAs) have been revealed in both cellular organisms and viruses. Micro (mi)RNAs (the best known ncRNAs) typically act to decrease the target mRNA level [8]. These transcripts are generated through the processing of long precursor RNA molecules. MicroRNAs have been detected in α-herpesviruses (HSV: [9], and PRV: [10,11]), betaherpesviruses (human cytomegalovirus (HCVM): [12]), and gammaherpesviruses (Epstein-Barr virus (EBV): [13]). These transcripts have been shown to play various roles, including the switch between the latent and lytic phases, evasion of host immune surveillance and apoptosis inhibition [14]. Long ncRNAs (lncRNAs) are the most abundant group of ncRNAs [15]. Numerous protein-encoding genes have been shown to specify antisense (as)-lncRNAs transcribed from the complementary DNA strands as templates. Large proportions of the mouse and human genomes have recently been reported to express lncRNAs [16,17]. The functions of these transcripts are still largely unknown. Many lncRNAs are involved in the regulation of transcription, such as *XIST* [18] and *HOTAIR* [19], or post-transcriptional regulation [20], or have structural roles [17]. Studies of multiple model systems have revealed that lncRNAs can function as modular scaffolds, forming extensive networks between chromatin regulators and various ribonucleoproteins [21]. Several polyadenylated lncRNAs have recently been demonstrated to be highly abundant in herpesviruses, including RNA2.7 in HCMV, accounting for nearly half of the total gene expression in RNA-Seq studies [22], and the widely-studied PAN RNA in Kaposi’s sarcoma-associated herpesvirus [23], which has diverse roles during the viral life cycle [24]. The HSV latency-associated transcript (LAT) was the first identified as-lncRNA molecule [25] in alphaherpesviruses. A spliced 8.4-kb RNA, termed the long latency transcript (LLT), is generated from the complementary DNA strand of *ie180* and *ep0* genes under the control of the LAT promoter of PRV [26]. The expression of as-lncRNAs has also been detected in some other HSV genes [27,28,29]. Moreover, several antisense long non-coding transcripts have been discovered in HCMV [30] and EBV [31].

## 2. Materials and Methods

### 2.1. Cells and Viruses

An immortalized porcine kidney epithelial cell line (PK-15) was used for the propagation of PRV. The cells were cultivated in Dulbecco’s modified Eagle medium supplemented with 5% fetal bovine serum (Gibco Invitrogen, Carlsbad, CA, USA) and 80 μg gentamycin/mL at 37 °C under 5% CO_2_. The virus stock used for the kinetic analyses was prepared as follows: rapidly-growing semi-confluent PK-15 cells were infected at a multiplicity of infection (MOI) of 0.1 pfu/cell, and then incubated at 37 °C under 5% CO_2_ until a complete cytopathic effect was observed. The infected cells were next frozen and thawed three times, followed by centrifugation at 10,000× *g* for 15 min. The titer of the virus stock was determined by using the same cell type. For the transcription kinetic experiments, cells were infected at either a low (0.1 pfu/cell) or a high MOI (10 pfu/cell), and then incubated for 1 h. This was followed by removal of the virus suspension and washing with phosphate-buffered saline. Infected cells were incubated for various periods of time following the addition of new medium to the cells.

For Illumina DNA sequencing we mixed infected cells, which were incubated for 1, 2, 4, 6, 8, 10, 12, 14, 16, 18, 20, 22 or 24 h. For PacBio analysis, infected cells were incubated 1, 2, 4, 6, 8 or 12 h p.i. For Real-Time RT-PCR, infected PK-15 cells were incubated for 1, 2, 4, 6, 8, 12 or 24 h. Mock-infected cells, which were otherwise treated in the same way as the infected cells, were used as controls.

### 2.2. Generation of Recombinant Viruses

The generation of *ep0* and *vhs* gene-deleted viruses was described elsewhere (*vhs-*KO: [32], *ep0-*KO: [33]). Briefly, the desired viral genes were deleted by targeted mutagenesis using homologous recombination. Following subcloning of the target region of PRV, a *lacZ* gene expression-cassette was inserted in place of the genes to be deleted in both mutants. Mutant viruses were selected on the basis of the blue plaque phenotype.

### 2.3. RNA Isolation for RNA-Seq and Real-Time RT-PCR

Total RNA was purified by using the Nucleospin RNA kit (Macherey-Nagel), following the kit protocol. Cells were collected by low-speed centrifugation, lysed in a buffer containing the chaotropic ions needed for the inactivation of RNases and providing the conditions for the binding of nucleic acids to a silica membrane. Contaminating DNA was removed with RNase-free rDNase solution (included in the kit). The isolated total RNA was treated by means of the TURBO DNA-free™ Kit (Life Technologies) to remove potential residual DNA contamination. RNA concentration was determined by Qubit 2.0, and RNA integrity was assessed by using an Agilent 2100 Bioanalyzer. Samples were stored at −80 °C.

### 2.4. Illumina HiScanSQ cDNA Sequencing

*Preparation of cDNA libraries*—strand-specific total RNA libraries were prepared for sequencing through use of the Illumina ScriptSeq v2 RNA-Seq Library Preparation Kit (Epicentre, Madison, WY USA) for random hexamer primed amplification and the sequencing of 2 × 100 bp fragments. For PA-Seq, a single-end library was constructed by using custom-anchored adaptor-primer oligonucleotides with an oligo(VN)T_20_ primer sequence. Anchored primers compensate for the loss in throughput due to the high fraction of reads containing solely adenine bases when conventional oligo(dT) primers are used.

Transcriptome sequencing was performed on an Illumina HiScanSQ platform, generating ~200 million paired-end reads of 100 bp length and ~105 million 50 bp single-end reads. The quality assessment of the raw read files was achieved with FastQC v0.10.1. Reads were aligned to the respective host genome (*Sus scrofa*, assembly: Sscrofa10.2) and subsequently to the PRV genome (KJ717942.1) by using Tophat v2.09. [34]; ambiguous reads were discarded. For PA-Seq, mapping was carried out with Bowtie v2. [35], and polyA peaks were detected through the use of in-house scripts, based on the criteria of the presence of a PA signal in the 50 bp region upstream from the PA site and the presence of at least two consecutive adenine mismatches in at least 10 independent reads at the PA site. Annotation and visualization were carried out with the Artemis Genome Browser v15.0.0 [36]. Any GC bias of the alignments was inspected with the Bioconductor R package.

### 2.5. PacBio RS II cDNA Sequencing

#### 2.5.1. PolyA RNA Purification

Polyadenylated RNAs were isolated from the total RNA samples by using the Oligotex mRNA Mini Kit (Qiagen, Venlo, The Netherlands) according to the kit instructions for the Oligotex mRNA Spin-Column Protocol.

#### 2.5.2. cDNA Synthesis

The PolyA RNA samples were quantified with the Qubit RNA HS Assay Kit (Life Technologies, Carlsbad, CA, USA) and converted to cDNAs with the SuperScript Double-Stranded cDNA Synthesis Kit (Life Technologies). RT reactions were primed with an Anchored Oligo(dT)_20_ primer (Life Technologies). The cDNAs were quantified with the Qubit HS dsDNA Assay Kit (Life Technologies) and quality was assessed with the Agilent 2100 bioanalyzer.

#### 2.5.3. Library Preparation, Sequencing and Data Collection

SMRTbell libraries were generated by using the PacBio DNA Template Prep Kit 2.0 and the Pacific Biosciences template preparation and sequencing protocol for Very Low (10 ng) Input 2 kb libraries with carrier DNA (pBR322, Thermo Scientific, Waltham, MA, USA). SMRTbell templates were bound to polymerases by using the DNA polymerase binding kit XL 1.0 (part #100-150-800) and v2 primers.

Polymerase-template complexes were bound to magbeads with the Pacific Biosciences MagBead Binding Kit, and sequencing was carried out on the Pacific Biosciences RSII sequencer with C3 sequencing reagents. Movie lengths were 180 min (one movie was recorded for each SMRT Cell). Subread filtering and alignment were carried out in SMRT Pipe v2.2.0. Visualization and data analysis were performed in SMRT Analysis v2.2.0.

### 2.6. Normalization of PacBio Data with Mitochondrial Transcripts

The read counts of viral transcripts at each time-point were normalized to mitochondrial read counts, aligned to the *Sus scrofa* 10.2 MT chromosome sequence. The following mitochondrial genes were used for the normalization: ATP6; ATP8; CYTB; ND1; ND2; ND3; ND4; ND4L; ND5; ND6; COX1; COX2 and COX3. While the degradation of cytoplasmic mRNAs during alphaherpesvirus infection has been previously shown [37,38], no such evidence is known for mtRNAs. Although recent studies have shown the steady decrease of mtDNA levels in Vero cells expressing the UL12.5 gene of HSV-1 [39]. We chose the mtRNAs as reference RNAs because the UL12.5 gene is absent from the PRV genome.

### 2.7. Reverse Transcription

RT reactions were carried out with 70 ng of total RNA with the use of Superscript III enzyme (Life Technologies) and gene-specific primers or oligo(dT) primers.

### 2.8. Real-Time PCR

Real-Time PCR reactions were performed in a volume of 20 µL with Absolute QPCR SYBR Green Mix (Thermo Scientific) containing 7 µL of 10-fold diluted cDNA, 1.5 μL of forward and 1.5 μL of reverse primers (10 μM each; Table 1A). 28S ribosomal (r)RNA was used as a reference gene in each run. The PCR amplification conditions were as follows: 15 min at 95 °C for the enzyme activation, followed by 30 cycles of 94 °C for 25 s (denaturation), 60 °C for 25 s (annealing), and 72 °C for 6 s (extension).

**Table 1:** Primer sequences for the Real-Time RT PCR analysis.

	Name	Sequence (5′-3′)	Genomic Position
A	CTO-S fw	GACGATCCGGCGGTCCCA	63858–63875
	CTO-S rev	GCGCCACAACCCGGAGC	63915–63931
	CTO-L fw	GTG TCG CGG ACA GAG AAT GG	64604–64623
	CTO-L rev	GGC CCA GTA CCT GTT TCA GC	64708–64727
B	T7-CTO-out fw	TAATACGACTCACTATAGGGAGAGGTCTCTAAGGGGGAACCAG	63605–63626
	SP6-CTO-out rev	ATTTAGGTGACACTATAGAAGNGCCGAAAAATTCGCACATACC	63989–64008

(underline: T7 and SP6 promoter sequences, respectively).

Relative expression ratios (*R*) were calculated via the following formula:

(1) $$R=\frac{{\left(E_{\text{sample}\cdot \text{max}}\right)}^{C{\text{t}}_{\text{sample}\cdot \text{max}}}}{{\left(E_{\text{sample}}\right)}^{C{\text{t}}_{\text{sample}}}}:\frac{{\left(E_{\text{ref}\cdot \text{max}}\right)}^{C{\text{t}}_{\text{ref}\cdot \text{max}}}}{{\left(E_{\text{ref}}\right)}^{C{\text{t}}_{\text{ref}}}}$$

where *E* is the amplification efficiency, *C*t is the threshold cycle number, “sample” refers to the examined PRV transcript and “ref” refers to the 28S rRNA (internal control). The cDNAs were normalized to 28S cDNAs by using the Comparative Quantitation module of the Rotor-Gene Q software (Version 2.3.1, Qiagen), which automatically calculates the efficiency of the reaction. Thresholds were also set by the software.

### 2.9. Treatment of Cells with CHX

The requirement of *de novo* protein synthesis for CTO production was tested by cycloheximide (CHX) analysis. Cells were incubated in the presence or absence of 100 μg/mL CHX (Sigma-Aldrich, St. Louis, MO, USA) for 1 h prior to virus infection. Mock-infected cells otherwise treated in the same way as infected cells were used as controls.

### 2.10. Northern Blot Analysis

*Traditional Northern blot assay* Total RNA was isolated from PK-15 cells through use of TRIzol reagent (Life Technologies) according to the manufacturer’s instructions. Samples were denatured in loading buffer for 5 min at 65 °C. Extracted RNA samples (10 ug) were fractionated in formaldehyde/1.2% agarose gel, transferred to a Nytran N membrane (Schleicher & Schuell BioScience, Dassel, Germany) by a capillary method and fixed by ultraviolet cross-linking. The membrane was probed by using the random primed PCR product or the total viral DNA with the DecaLabel DNA Labeling Kit (Fermentas, Vilnius, Lithuania). PCR reactions were carried out with AccuPrime GC-Rich DNA Polymerase (Life Technologies) according to the manufacturer’s recommendations (primer sequences Table 1B). The oligonucleotide probe was labeled with [α-^32^P]CTP. Filter prehybridization was carried out in 50% formamide, 0.5% SDS, 5× SSPE, 5× Denhardt’s solution and 20 μg/mL sheared, denatured salmon sperm. The probe was heated for 1 min at 95 °C. Overnight hybridization was carried out at 68 °C. Finally, the hybridization membranes were washed in 2× SSC 0.1% SDS at 68 °C for ones 10 min, 0.5× SSC, 0.1% SDS at 68 °C for 10 min, 0.1× SSC 0.1% SDS at 68 °C for 10 min.

*Micro RNA Northern blot analysis* two different PCR probes were used. Forward primers were linked with the T7 promoter sequence and reverse primers were linked with the SP6 promoter sequence (Table 1B). Samples (10 µg) were fractionated on denaturing 12% polyacrylamide gels containing 8 M urea, transferred to a Nytran N membrane (Schleicher & Schuell, Germany) by a capillary method and fixed by ultraviolet cross-linking. Prehybridization was carried out in 50% formamide, 0.5% SDS, 5× SSPE, 5× Denhardt’s solution and 20 μg/mL sheared, denatured salmon sperm DNA. Overnight hybridizations were performed in the same solution at 37 °C. An [α-^32^P]UTP-labeled RNA probe was used for the hybridization. Membranes were washed twice for 10 min with a solution containing 2× SSC, 0.1% SDS.

## 3. Results

### 3.1. Identification and Structural Characterization of Novel lncRNAs in PRV

**Figure 1:**
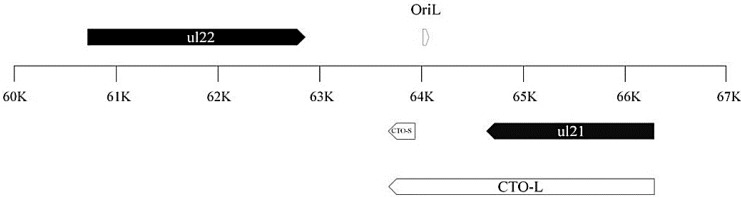
Location of *cto* genes on the PRV genome. Both *cto* transcripts (CTOs) are polyadenylated RNAs with a common 3′ termination. CTO-L is generated by the continuation of transcription after the termination signals of the *ul21* gene. OriL is the replication origin of in the UL region of viral DNA mapped between the *ul21* and *ul22* genes.

The PRV transcriptome was analyzed by means of the Illumina HiScanSQ and Pacific Biosciences (PacBio) RSII sequencing systems. Random hexamer-primed reverse transcription (RT) was used for Illumina sequencing, and oligo(dT)-primed (PA-Seq) RT for both platforms. With these techniques, we detected two novel 3′-coterminal transcripts located between the *ul21* and *ul22* genes, close to the OriL, termed CTOs. The length of the short intergenic lncRNA (CTO-S) is 286 base pairs (bp) and is mapped to bp-s 63673-63958 of the PRV reference genome KJ717942.1 (Figure 1). The attachment of adapter sequences to the Illumina RT primers allowed the analysis of transcription from both DNA strands separately. These investigations revealed that only one of the two DNA strands exhibits transcriptional activity at this genomic region. The long (CTO-L) transcript overlaps OriL, and maps to nucleotides (nt) 63673–66287 (2615 bp). CTO-L originates from the promoter of the *ul21* gene and is produced by the continuation of the RNA polymerase molecule across the transcription termination sequences. CTO-L contains the entire *ul21* gene sequence and is therefore a sense lncRNA. The promoter of the CTO-S transcript was identified in nucleotides 63952–63958 by the Tfsearch algorithm with 96.8% confidence. An Oct1 transcription factor binding site was also discovered at 98.3% confidence in the TransFac database [40].

### 3.2. Transcriptional Analysis of CTO

#### 3.2.1. Illumina RNA-Seq Analysis

We combined transcripts isolated from consecutive time-points of viral infection for Illumina sequencing. CTO-S proved to exhibit a very high expression, with an RPKM (reads per kilobase per million) value of 1.6 × 10^6^ in the random-hexamer primed library, and 45.9% of the total read count in PA-Seq, making this transcript by far the most abundant viral RNA molecule. CTO-L produced only 0.13% of the total reads in the random hexamer-primed library (RPKM = 5 × 10^−4^). However, pA-Seq produces more informative data than random hexamer-primed sequencing: in the former case the read numbers are in strict correlation only with the transcript abundance, whereas in the latter case they correlate with the transcript lengths too.

#### 3.2.2. PacBio RNA-Seq Analysis

**Figure 2:**
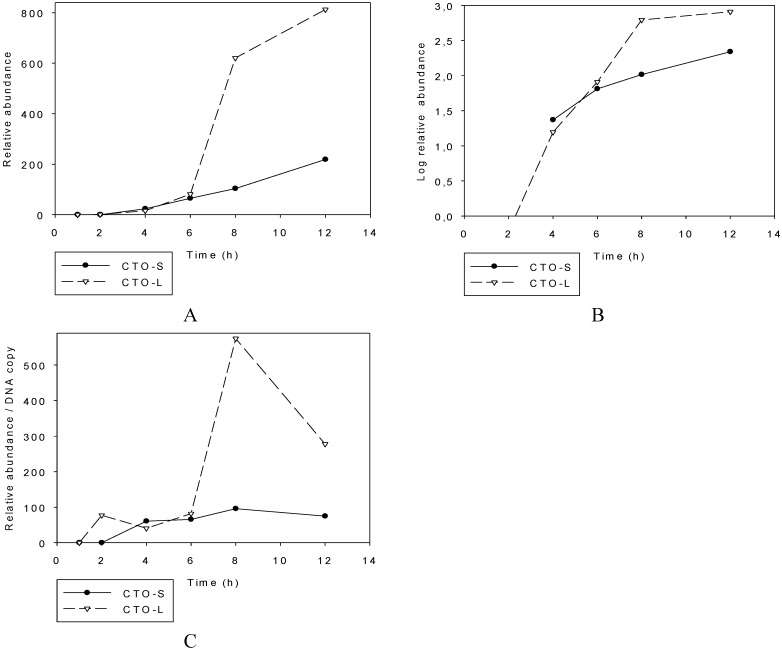
Transcription kinetics of CTO transcripts. The relative abundances of transcripts are depicted on a linear (**A**) and a logarithmic (**B**) scale. All RNA reads obtained by PacBio sequencing were normalized with mitochondrial RNAs. The transcriptional activity of the CTOs was also analyzed by normalizing the data with the relative amount of viral DNAs (**C**).

For the analysis of the transcription kinetics of the CTO length variants, we applied the PacBio RS II system, which is capable of generating significantly longer read lengths than those of second-generation technologies, such as Illumina. The CTO expression was analyzed at 1, 2, 4, 6, 8 or 12 h by using high [] infection conditions. Due to template quantity we used the very low input protocol for the template preparation and sequencing, which is not optimal for the detection of small (<700 nt) transcripts, and we therefore observed a very strong bias against CTO-S. Due to the low sensitivity of this technique for small DNA fragments, the real proportion of the two transcripts cannot be precisely ascertained through its use. However, the data obtained could be used to compare the transcription kinetics in the two transcripts (Figure 2). The viral RNA reads were normalized with the pig mitochondrial RNAs, which are thought to resist degradation by the RNase activity of viral proteins. No reads were obtained for either of the CTO transcripts in the first hour of infection. A low amount of CTO-L was detected 2 h post-infection (p.i.). The CTO-S transcript appeared in only the 4 h p.i. samples (Figure 2A). The logarithmic plots demonstrate a slight increase in the dynamics of transcriptions between 4 and 6 h p.i. in both transcripts (Figure 2B), followed by an elevated expression rate, especially in CTO-L, which increased very steeply after 6 h p.i.. However, analysis of the transcriptional activity normalized to the copy number of PRV DNA (determined by Real-Time RT-PCR) demonstrated that the expression from individual DNA molecules was highest at 8 h p.i. for both transcripts (Figure 2C).

**Figure 3:**
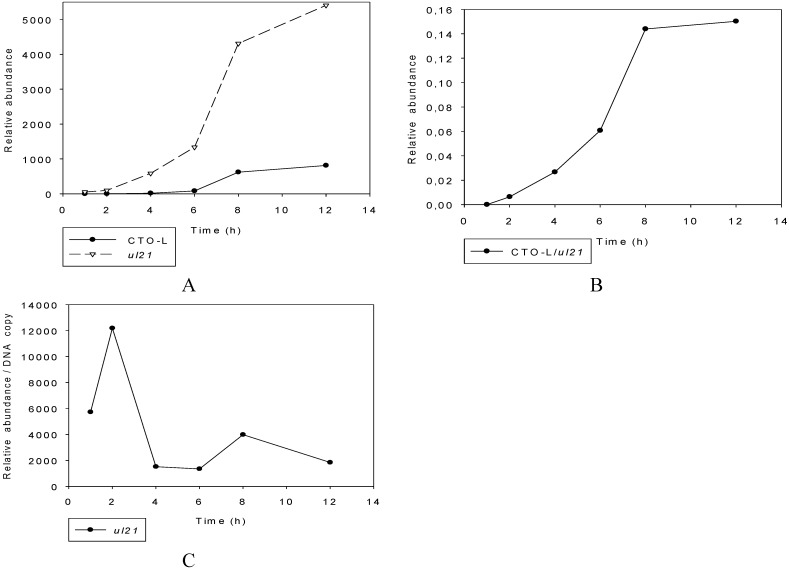
Comparison of the amounts of ul21 and CTO-L transcripts. CTO-L is a readthrough product of the *ul21* gene. (**A**) shows the transcriptional kinetics of the two transcripts, while (**B**) shows the change in readthrough efficiency with time. All RNA reads obtained by PacBio sequencing were normalized with mitochondrial RNAs. (**C**) shows the transcriptional activity normalized to the viral genome.

We examined whether the efficiency of transcriptional readthrough varied in time by comparing the amounts of CTO-L and ul21 mRNA (Figure 3A,B). The data revealed that the ratio of CTO-L to the ul21 transcript increased continuously in time. An examination as to whether this was simply due to a higher transcription rate of individual *ul21* genes did not indicate an ycorrelation between the readthrough efficiency and the transcriptional activity of this gene when the transcript reads were normalized with the DNA copy number (Figure 3C). This suggests that the efficiency of the recognition of transcriptional termination sequences might be regulated by a specialized mechanism.

#### 3.2.3. Multi-Time-Point Real-Time RT-PCR Analysis of CTOs in Wild-Type (wt) and Mutant Backgrounds

##### Wild-type PRV

**Figure 4:**
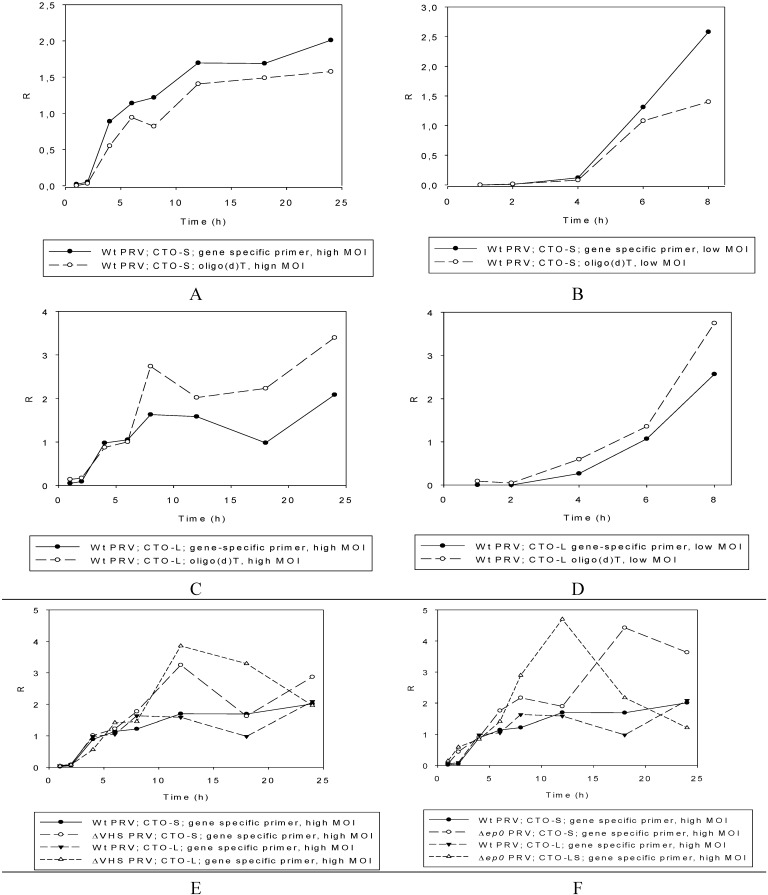
The change in relative expression ratio (R) of the CTO transcripts with time, determined by Real-Time RTR-PCR. (**A**). CTO-S: High-titer (10 pfu/cell) infection. The cDNAs were generated by reverse transcription of CTO-S transcripts through the use of gene-specific or oligo(dT) primers. (**B**). CTO-S: Low-titer (0.1 pfu/cell) infection. The cDNAs were generated by reverse transcription of CTO-S transcripts through the use of gene-specific or oligo(dT) primers. (**C**). CTO-L: High-titer (10 pfu/cell) infection. The cDNAs were generated by reverse transcription of CTO-S transcripts through the use of gene-specific or oligo(dT) primers. (**D**). CTO-L: Low-titer (0.1 pfu/cell) infection. The cDNAs were generated by reverse transcription of CTO-S transcripts through the use of gene-specific or oligo(dT) primers. (**E**). Expression of CTO-S in *vhs-KO* background following high-titer infection. (**F**). Expression of CTO-S in *ep0-KO* background following high-titer infection.

Strand-specific priming-based RT was used for the kinetic assay of the abundant CTO-S transcript in both low-titer (0.1 pfu/cell) and high-titer (10 pfu/cell) infection. The method used for the calculation of relative expression ratios (R) was as described earlier [41]. Real-Time RT-PCR analyses confirmed the PacBio and Illumina RNA-Seq results, showing that practically no transcription occurred in the first 2 h of the viral life cycle in the genomic region encoding CTO-S (Figure 3A). In the high-titer infection, CTO-S reached very high levels by 4 h p.i. (Figure 4A), which means that the expression of this transcript is initiated sometime between 2 and 4 h p.i. In the low-titer experiment, however, CTO-S was expressed at a very low level at 4 h, but reached a high level by 6 h p.i. (Figure 4B). Thus, there is a shift in the expression kinetics of CTO-S transcripts in low-pfu as compared with high-pfu experiments. The CTO-L expression was examined by using strand-specific primers for the reverse transcriptions at 1, 2, 4, 6, 8, 12, 18 and 24 h at high-titer infection (Figure 4C) and 1, 2, 4, 6 and 8 h at low-titer infection (Figure 4D). There was no significant expression until 4 h p.i., which confirmed the PacBio sequencing data.

##### Mutant PRVs

Two mutant viruses were used to analyze the CTO-S and CTO-L transcription kinetics in order to evaluate the potential effects of mutations on the expression kinetics of this transcript. The levels of the two transcripts were higher than that of the wt virus in the *vhs-KO* virus (Figure 4E), which is not surprising since the virion host shut-off (VHS) protein plays a role in the destabilization of RNA molecules [42]. We earlier reported similar transcription kinetics for the rest of the PRV genes [32]. The expression kinetics of the CTOs in the *ep0-KO* (ep0: early protein 0) background, however, exhibits an atypical pattern since the transcript levels of other late genes of the wt virus are generally higher than those of the *ep0*-null mutant virus, which is not the case for these lncRNAs (Figure 4F). Additionally, in contrast to the wt virus, the level of CTO-S and CTO-L is relatively high at 2 h p.i. in this mutant virus. Thus, EP0 appears to exert a down-regulatory effect on the transcription of CTOs throughout the whole life cycle of the virus.

### 3.3. CTO Expression is Controlled by the IE180 Transactivator of PRV

**Figure 5:**
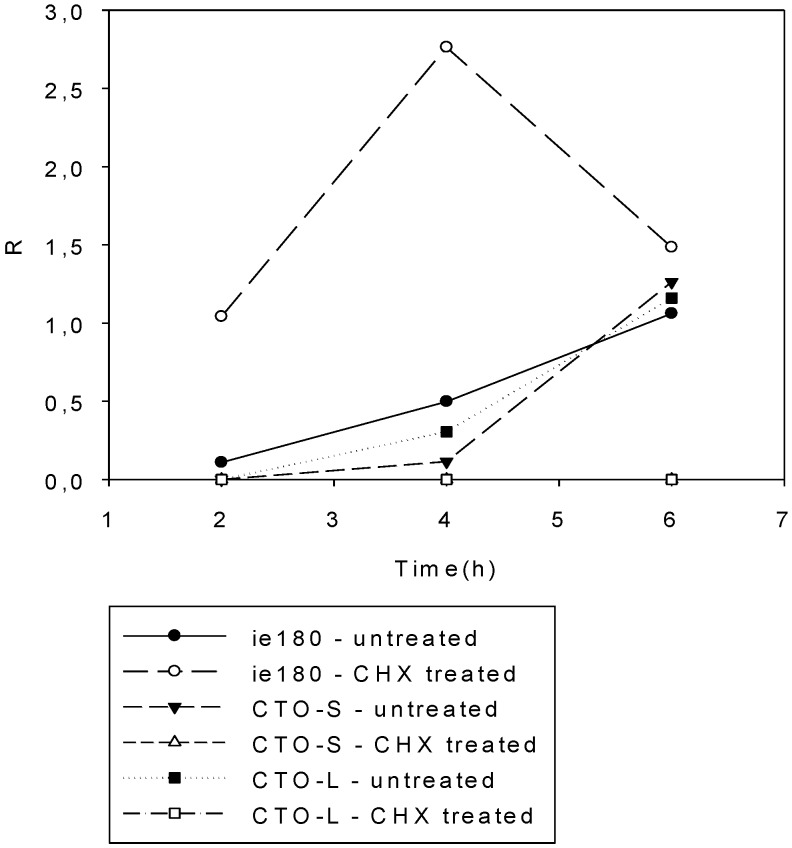
Analysis of transcription following CHX treatment of infected cells. The *ie180* gene is not repressed by cycloheximide (CHX), but CTO-S is totally blocked by this protein synthesis inhibitor, the reason for this being that CTO-S requires the IE180 protein for its expression.

The transcription of PRV genes is controlled by the IE180 transactivator protein. Cycloheximide (CHX), an inhibitor of protein synthesis in eukaryotic cells, completely blocked gene expression, except in the *ie180* gene itself and two as-lncRNA-encoding genes, LAT and LLT. The repression of CTO expression in the presence of CHX indicates that the transcription of these molecules is fully dependent on IE180 (Figure 5). Earlier we had shown that *ul21* gene expression was completely repressed by CHX treatment [41], which—due to their sharing a common promoter—also resulted in the silencing of CTO-L transcription.

### 3.4. CTO Expression in the Presence of an Inhibitor of DNA Replication

We additionally investigated the effect of phosphonoacetic acid (PAA), an inhibitor of DNA synthesis, on the transcription kinetics of CTO-S. The method of calculation for the evaluation of the repressive effect of PAA on the transcription of the individual genes was published earlier [41]: R_i-PAA_ = R_6h-PAA_/R_6h-UT_. In the present study, the average R_i-PAA_ values were found to be 0.717 for early genes and 0.113 for late genes. The value of R_i-PAA_ = 0.184 for CTO-S and R_i-PAA_ = 0.361 for CTO-L confirmed the result of our kinetic analyses in non-treated samples: these transcripts are expressed with late kinetics.

### 3.5. Northern Blot and *in Silico* Analyses Revealed that CTO-S is not a miRNA Precursor

Our investigation of whether CTO-S might be a miRNA precursor by using microRNA Northern blot analysis, however, did not detect any transcript with miRNA size in this genomic region (data not shown). Conversely, CTO-S RNA was detected by using traditional Northern blot analysis (Figure S1). Due to the very low copy number, we could not detect CTO-L by Northern blot analysis, however, the existence of this transcript was verified by four independent techniques (PacBio PA-seq, two Illumina RNA-seq methods and Real-time RT-PCR). Sequence analysis of CTO by using the pre-microRNA hairpin prediction tools miRNAFold [43] and miPred [44] yielded negative results in each case. Moreover, previous studies of the miRNA expression in PRV in both porcine dendritic [10] and epithelial [11] cell lines failed to detect miRNAs from the genomic region of CTO.

## 4. Discussion

**Figure 6:**
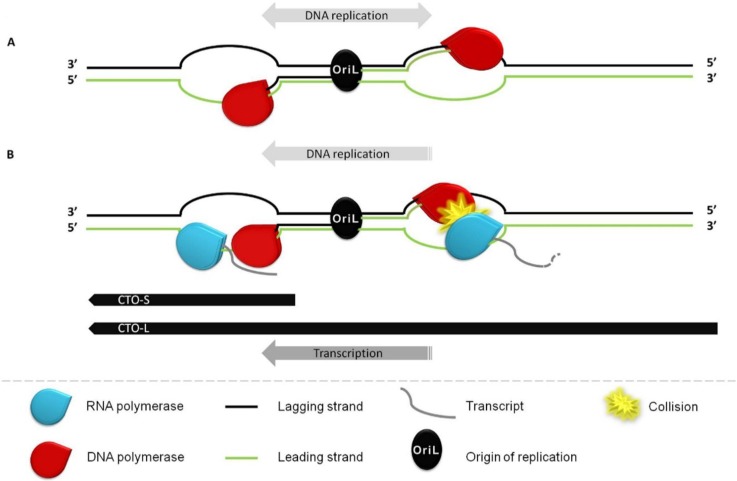
A proposed model for the interactions between the replication and transcription machineries. (**A**) There is no CTO expression in the early stage of infection, and this allows the bidirectional synthesis of DNA (δ type replication). (**B**) Later, the transcription machineries of the CTOs facilitate unidirectional DNA replication through two mechanisms: (1) the DNA polymerase collides with the RNA polymerase synthesizing CTO-L, thereby halting the progression and/or preventing the assembly of the replication machinery (right to OriL); and (2) RNA polymerase transcribing the CTO-S (or CTO-L) facilitates the progression of DNA polymerase in one way through unwinding of the two DNA strands (left to OriL). For the sake of simplicity, the DNA synthesis from the lagging strand is not depicted.

In this study we report the identification and characterization of two lncRNAs of pseudorabies virus. The two transcripts share a common poly(A) signal. CTO-S, a short intergenic lncRNA molecule is found to be very abundant, but is not expressed in the first 2 h of viral infection. We have demonstrated that the expression of CTO-S is controlled by the virally-encoded IE180 transactivator. CTO-L is expressed at a relatively low level, produced from the promoter of the *ul21* gene through occasional transcriptional readthrough events across the transcription termination signal of this gene. The levels of CTO-S transcripts in the mutant viruses are higher at every time point than in the wt PRV, indicating a role of these gene products in the stability and/or the regulation of these molecules at the level of transcription. The vicinity of CTO-S to OriL and the overlap of CTO-L with OriL suggest a role of this genomic region in the regulation of DNA replication, which may be based on the interference between the transcriptional and replication machineries, as suggested by Huvet *et al*. [45]. Others did not verify the Huvet model, at least in human cells [46]. Despite this, interference between the two apparatuses may be an existing mechanism; the RNA polymerase molecules transcribing CTO-L might clash with DNA polymerase, thereby preventing the progress of replication in one of the two directions (Figure 6). It has been hypothesized, but never proved, that the synthesis of alphaherpesvirus DNAs starts with δ-type replication, which is followed by a switch to sigma-type replication generating concatemers [47]. The transcription of CTO-S may facilitate replication in another way, through separation of the two DNA strands, thereby helping the progression of DNA polymerase in one direction. In this scenario, the lack of CTO expression in the first few hours of the viral life cycle allows bidirectional δ-type replication; later, the process of CTO transcription itself makes the replication unidirectional through the two mechanisms proposed above. If there is no δ-type replication, and the viral DNA synthesis starts with CTO transcription itself, this makes the replication unidirectional through the two mechanisms proposed above. If there is no δ-type replication, and the viral DNA synthesis starts with concatemer formation immediately, the above putative mechanism might also contribute to the unidirectionality of DNA synthesis. Overall, extensive transcriptional activity near oriL potentially exerts an effect on the DNA replication by determining the orientation of the DNA synthesis and perhaps contributing to the switch from bidirectional to unidirectional replication.

The polyadenylation of CTO-S indicates that this transcript may have additional function(s) in the life cycle of the virus. These transcripts do not have an essential role in the viral replication since two strains (TJ [48] and ZJ01 [49]) contain deletions at this genomic region.

In this putative mechanism, the transcripts are merely by-products. It has been shown in a variety of organisms that a similar mechanism based on the clash between two RNA polymerase molecules in the overlapping region may have a regulatory effect on the transcription through transcriptional interference [50,51,52].

## Supplementary Materials

Supplementary materials can be found at http://www.mdpi.com/1999-4915/7/5/2727/s1.

## References

[1] Pomeranz L.E., Reynolds A.E., Hengartner C.J. (2005). Molecular biology of pseudorabies virus: Impact on neurovirology and veterinary medicine. Microbiol. Mol. Biol. Rev..

[2] Szpara M.L., Kobiler O., Enquist L.W. (2010). A common neuronal response to alphaherpesvirus infection. J. Neuroimmune Pharmacol..

[3] Strack A.M. (1994). Pseudorabies virus as a transneuronal tract tracing tool: Specificity and applications to the sympathetic nervous system. Gene Ther..

[4] Card J.P., Enquist L.W. (2001). Transneuronal circuit analysis with pseudorabies viruses. Curr. Protoc. Neurosci..

[5] Boldogkői Z., Sík A., Dénes A., Reichart A., Toldi J., Gerendai I., Kovács K.J., Palkovits M. (2004). Novel tracing paradigms—Genetically engineered herpesviruses as tools for mapping functional circuits within the CNS: Present status and future prospects. Prog. Neurobiol..

[6] Boldogkői Z., Bálint K., Awatramani G.B., Balya D., Busskamp V., Viney T.J., Lagali P.S., Duebel J., Pásti E., Tombácz D. (2009). Genetically timed, Activity sensor and Rainbow transsynaptic viral tools. Nat. Methods.

[7] Prorok J., Kovács P.P., Kristóf A.A., Nagy N., Tombácz D., Tóth J.S., Ördög B., Jost N., Virág L., Papp J.G. (2009). Herpesvirus-mediated delivery of a genetically encoded fluorescent Ca^2+^ sensor to canine cardiomyocytes. J. Biomed. Biotechnol..

[8] Janowski B.A., Kaihatsu K., Huffman K.E., Schwartz J.C., Ram R., Hardy D., Mendelson C.R., Corey D.R. (2005). Inhibiting transcription of chromosomal DNA with antigene peptide nucleic acids. Nat. Chem. Biol..

[9] Umbach J.L., Kramer M.F., Jurak I., Karnowski H.W., Coen D.M., Cullen B.R. (2008). MicroRNAs expressed by herpes simplex virus 1 during latent infection regulate viral mRNAs. Nature.

[10] Anselmo A., Flori L., Jaffrezic F., Rutigliano T., Cecere M., Cortes-Perez N., Lefèvre F., Rogel-Gaillard C., Giuffra E. (2011). Co-expression of host and viral microRNAs in porcine dendritic cells infected by the pseudorabies virus. PLoS ONE.

[11] Wu Y.Q., Chen D.J., He H.B., Chen D.S., Chen L.L., Chen H.C., Liu Z.F. (2012). Pseudorabies virus infected porcine epithelial cell line generates a diverse set of host microRNAs and a special cluster of viral microRNAs. PLoS ONE.

[12] Grey F., Antoniewicz A., Allen E., Saugstad J., McShea A., Carrington J.C., Nelson J. (2005). Identification and characterization of human cytomegalovirus-encoded microRNAs. J. Virol..

[13] Pfeffer S., Zavolan M., Grässer F.A., Chien M., Russo J.J., Ju J., John B., Enright A.J., Marks D., Sander C., Tuschl T. (2004). Identification of virus-encoded microRNAs. Science.

[14] Li K., Ramchandran R. (2010). Natural antisense transcript: A concomitant engagement with protein-coding transcript. Oncotarget.

[15] Mattick J.S., Makunin I.V. (2006). Non-coding RNA. Hum. Mol. Genet..

[16] Carninci P., Kasukawa T., Katayama S., Gough J., Frith M.C., Maeda N., Oyama R., Ravasi T., Lenhard B., Wells C. (2005). The transcriptional landscape of the mammalian genome. Science.

[17] Wilusz J.E., Sunwoo H., Spector D.L. (2009). Long noncoding RNAs: Functional surprises from the RNA world. Genes Dev..

[18] Zhao J., Sun B.K., Erwin J.A., Song J.J., Lee J.T. (2008). Polycomb proteins targeted by a short repeat RNA to the mouse X chromosome. Science.

[19] Tsai M.C., Manor O., Wan Y., Mosammaparast N., Wang J.K., Lan F., Shi Y., Segal E., Chang H.Y. (2010). Long noncoding RNA as modular scaffold of histone modification complexes. Science.

[20] Tripathi V., Ellis J.D., Shen Z., Song D.Y., Pan Q., Watt A.T., Freier S.M., Bennett C.F., Sharma A., Bubulya P.A. (2010). The nuclear-retained noncoding RNA MALAT1 regulates alternative splicing by modulating SR splicing factor phosphorylation. Mol. Cell..

[21] Rinn J.L., Chang H.Y. (2012). Genome regulation by long noncoding RNAs. Annu. Rev. Biochem..

[22] Gatherer D., Seirafian S., Cunningham C., Holton M., Dargan D.J., Baluchova K., Hector R.D., Galbraith J., Herzyk P., Wilkinson G.W. (2011). High-resolution human cytomegalovirus transcriptome. Proc. Natl. Acad. Sci. USA.

[23] Sun R., Lin S.F., Gradoville L., Miller G. (1996). Polyadenylylated nuclear RNA encoded by Kaposi sarcoma-associated herpesvirus. Proc. Natl. Acad. Sci. USA.

[24] Rossetto C.C., Tarrant-Elorza M., Verma S., Purushothaman P., Pari G.S. (2013). Regulation of viral and cellular gene expression by Kaposi’s sarcoma associated herpesvirus polyadenylated nuclear RNA. J. Virol..

[25] Stroop W.G., Rock D.L., Fraser N.W. (1984). Localization of herpes simplex virus in the trigeminal and olfactory systems of the mouse central nervous system during acute and latent infections by *in situ* hybridization. Lab. Investig..

[26] Cheung A.K. (1989). Detection of pseudorabies virus transcripts in trigeminal ganglia of latently infected swine. J. Virol..

[27] Ward P.L., Barker D.E., Roizman B. (1996). A novel herpes simplex virus 1 gene, *UL43.5*, maps antisense to the *UL43* gene and encodes a protein which colocalizes in nuclear structures with capsid proteins. J. Virol..

[28] Chang Y.E., Menotti L., Filatov F., Campadelli-Fiume G., Roizman B. (1998). *UL27.5* is a novel γ2 gene antisense to the herpes simplex virus 1 gene encoding glycoprotein B. J. Virol..

[29] Jovasevic V., Roizman B. (2010). The novel HSV-1 US5-1 RNA is transcribed off a domain encoding US5, US4, US3, US2 and α22. Virol. J..

[30] Zhang G., Raghavan B., Kotur M., Cheatham J., Sedmak D., Cook C., Waldman J., Trgovcich J. (2007). Antisense transcription in the human cytomegalovirus transcriptome. J. Virol..

[31] Iwakiri D., Takada K. (2010). Role of EBERs in the pathogenesis of EBV infection. Adv. Cancer Res..

[32] Tombácz D., Tóth J.S., Boldogkői Z. (2011). Deletion of the virion host shut: Off gene of pseudorabies virus results in selective upregulation of the expression of early viral genes in the late stage of infection. Genomics.

[33] Tombácz D., Tóth J.S., Boldogkoi Z. (2012). Effects of deletion of the early protein 0 gene of pseudorabies virus on the overall viral gene expression. Gene.

[34] Trapnell C., Pachter L., Salzberg S.L. (2009). TopHat: Discovering splice junctions with RNA-Seq. Bioinformatics.

[35] Langmead B., Salzberg S.L. (2012). Fast gapped-read alignment with Bowtie 2. Nat. Methods.

[36] Rutherford K., Parkhill K., Crook J., Horsnell T., Rice P., Rajandream M.A., Barrell B. (2000). Artemis: Sequence visualization and annotation. Bioinformatics.

[37] Kwong A.D., Frenkel N. (1989). The herpes simplex virus virion host shutoff function. J. Virol..

[38] Lin H.W., Chang Y.Y., Wong M.L., Lin J.W., Chang T.J. (2004). Functional analysis of virion host shutoff protein of pseudorabies virus. Virology.

[39] Saffran H.A., Pare J.M., Corcoran J.A., Weller S.K., Smiley J.R. (2007). Herpes simplex virus eliminates host mitochondrial DNA. EMBO Rep..

[40] Matys V., Kel-Margoulis O.V., Fricke E., Liebich I., Land S., Barre-Dirrie A., Reuter I., Chekmenev D., Krull M., Hornischer K. (2006). TRANSFAC and its module TRANSCompel: Transcriptional gene regulation in eukaryotes. Nucleic Acids Res..

[41] Tombácz D., Tóth J.S., Petrovszki P., Boldogkői Z. (2009). Whole-genome analysis of pseudorabies virus gene expression by real-time quantitative RT-PCR assay. BMC Genomics.

[42] Taddeo B., Roizman B. (2006). The virion host shutoff protein (UL41) of herpes simplex virus 1 is an endoribonuclease with a substrate specificity similar to that of RNase A. J. Virol..

[43] Tempel S., Tahi F. (2012). A fast ab-initio method for predicting miRNA precursors in genomes. Nucleic Acids Res..

[44] Jiang P., Wu H., Wang W., Ma W., Sun X., Lu Z. (2007). MiPred: Classification of real and pseudo microRNA precursors using random forest prediction model with combined features. Nucleic Acids Res..

[45] Huvet M., Nicolay S., Touchon M., Audit B., d’Aubenton Carafa Y., Arneodo A., Thermes C. (2007). Human gene organization driven by the coordination of replication and transcription. Genome Res..

[46] Necsulea A., Guillet C., Cadoret J.C., Prioleau M.N., Duret L. (2009). The relationship between DNA replication and human genome organization. Mol. Biol. Evol..

[47] Ward S.A., Weller S.K. (2011). HSV-1 DNA replication. Alpharpesviruses.

[48] Gu Z., Dong J., Wang J., Hou C., Sun H., Yang W., Bai J., Jiang P. (2015). A novel inactivated gE/gI deleted pseudorabies virus (PRV) vaccine completely protects pigs from an emerged variant PRV challenge. Virus Res..

[49] Luo Y., Li N., Cong X., Wang C.H., Du M., Li L., Zhao B., Yuan J., Liu D.D., Li S. (2014). Pathogenicity and genomic characterization of a pseudorabies virus variant isolated from Bartha-K61-vaccinated swine population in China. Vet. Microbiol..

[50] Osato N., Suzuki Y., Ikeo K., Gojobori T. (2007). Transcriptional interferences in cis natural antisense transcripts of humans and mice. Genetics.

[51] Gullerova M., Proudfoot N.J. (2012). Convergent transcription induces transcriptional gene silencing in fission yeast and mammalian cells. Nat. Struct. Mol. Biol..

[52] Boldogkői Z. (2012). Transcriptional interference networks coordinate the expression of functionally related genes clustered in the same genomic loci. Front. Genet..

