# Supplementary material for: Characterization of Novel Transcripts in Pseudorabies Virus
Source: Viruses. 2015 May 22;7(5):2727–44. doi: 10.3390/v7052727 (PMC4452928; doi:10.3390/v7052727)
Supplement: Supplementary file 1 [file viruses-07-02727-s001.pdf]

Supplementary Information

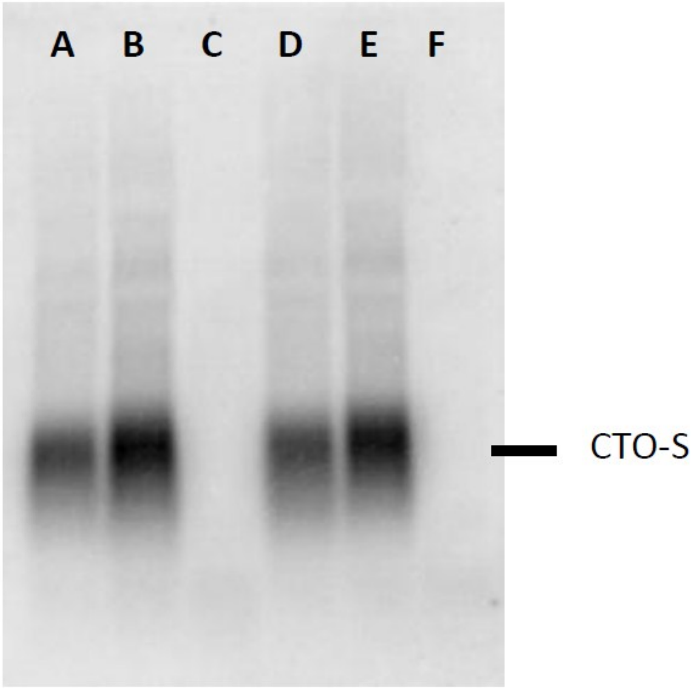

Figure S1. Northern blot analysis of the CTO-S transcript. The lanes were loaded with RNAs isolated from PK-15 cells 4 h p.i (*A* and *D*), 6 h p.i. (*B* and *E*), or non-infected (*C* and *F*). Lanes within the same brackets indicate parallel experiments. The ^{32}P-labeled antisense-RNA probes used for the hybridization were produced by *in vitro* transcription from PCR products containing the CTO-S lnc-RNA gene. The sense RNA was also used as a probe, but it yielded no hybridization, indicating that only one of the DNA strand of CTO-S region is transcriptionally active (data not shown).
